# Biomimetic synthesis of Ag-coated glasswing butterfly arrays as ultra-sensitive SERS substrates for efficient trace detection of pesticides

**DOI:** 10.3762/bjnano.10.59

**Published:** 2019-02-28

**Authors:** Guochao Shi, Mingli Wang, Yanying Zhu, Yuhong Wang, Xiaoya Yan, Xin Sun, Haijun Xu, Wanli Ma

**Affiliations:** 1 State Key Laboratory of Metastable Materials Science & Technology and Key Laboratory for Microstructural Material Physics of Hebei Province, School of Science, Yanshan University, Qinhuangdao, Hebei 066004, P.R. China; 2 Faculty of science, Beijing University of Chemical Technology, Beijing 100029, P.R. China; 3 Department of Mathematics, NC State University, Raleigh, NC 276968205, USA

**Keywords:** Ag nanofilm, glasswing butterfly, pesticide, surface-enhanced Raman scattering (SERS)

## Abstract

In this work, we report a biomimetic synthesis route of 3D Ag nanofilm/glasswing butterfly wing hybrids (Ag-G.b.) by magnetron sputtering technology. The 3D surface-enhanced Raman scattering (SERS) substrate is fabricated from an original chitin-based nanostructure, which serves as a bio-scaffold for Ag nanofilms to be coated on. The novel crisscrossing plate-like nanostructures of 3D Ag-G.b. nanohybrids with thick Ag nanofilms provide a substantial contribution to SERS enhancement. Measuring the SERS performance with crystal violet (CV), the Ag-G.b. nanohybrids with the sputtering time of 20 min (Ag-G.b.-20) shows the highest enhancement performance with an enhancement factor (EF) of up to 2.96 × 10^7^. The limit of detection (LOD) for CV was as low as 10^−11^ M, demonstrating the ultrahigh sensitivity of the Ag-G.b.-20 substrate. In addition, the Ag-G.b.-20 substrate has an outstanding reproducibility across the entire area with the maximum value of relative standard deviation (RSD) of less than 10.78%. The nanohybrids also exhibit a long-term stability regarding Raman enhancement, as suggested by a duration stability test over a period of 60 days. Importantly, the high-performance Ag-G.b.-20 substrate is further applied as an ultra-sensitive SERS platform for the trace detection of acephate, showing its great potential application in biochemical sensing and food security.

## Introduction

Surface-enhanced Raman scattering (SERS), an extension of conventional Raman spectroscopy, is a powerful analytical spectroscopy technique that can achieve single-molecule detection and provide high-resolution “fingerprint” spectral information [1–2]. The electromagnetic enhancement mechanism (EM), a consequence of the coherent collective electron oscillation, plays a dominant role in the enhancement of Raman signal intensity [3]. When incident light interacts with the free conduction electrons near the metallic plasmonic nanostructures, the collective oscillation of these electrons is significantly enhanced at metal–dielectric interfaces, which is known as localized surface plasmon resonance (LSPR). Namely, when a probe molecule is adsorbed in the vicinity of noble-metal (Au, Ag) nanostructures, its Raman signal intensity is enhanced by several orders of magnitude [4]. Besides from EM, chemical enhancement (CE) is the other widely recognized enhancement mechanism. In CE, the signal enhancement arises from a dynamic electron transfer between probe molecules and nanostructures. In contact with the nanostructures, the adsorbed molecules exhibit a larger scattering cross section, thus enhancing the Raman signal intensity efficiently [5]. However, CE only contributes to the enhancement factor (EF) up to 10^1^ to 10^2^ [6]. Therefore, in order to achieve high EF with outstanding sensitivity as well as reproducibility, the best strategy is to design optimal nanostructure that maximizes the LSPR effect.

According to previous reports [7–8], the lack of the SERS-active substrates with large-scale and high-density “hot spots” has become the biggest obstacle in the practical application of SERS detection. If a SERS substrate lacks dense “hot spots”, a long spectral acquisition time is required and the target species may be damaged. Two major approaches, wet-chemical or physical methods, are often adopted to develop SERS substrates. Metal plasmonic nanostructures with specific shapes such as Au nanorods [9], Au nanostars [10], Ag nanocubes [11], porous Au nanoparticles [12] and pyramidal Ag [13] have been successfully synthesized by wet-chemical approaches. These plasmonic nanostructures can be used as SERS substrates with low consumption and high EF. However, these synthesis methods are either difficult to control, have a low yield or require harsh experimental conditions as well as sophisticated instrumentation. Hence, a controllable and convenient way to fabricate high-performance SERS substrates would be invaluable in research and industrial application. Researchers have paid attention to physical methods (“top-down” techniques) such as focused ion beam lithography (FIB) [14–15], electron beam lithography (EBL) [16–17] or soft nanoimprint nanolithography (NIL) [18], which can produce controllable shapes and homogeneous plasmonic nanostructures. These physical methods, unfortunately, are limited by their high cost and time-consuming experimental processes. By using chemical methods (“bottom-up” techniques), Au or Ag nanoparticles were prepared to develop two- and three-dimensional nanostructures. However, these chemical methods cannot avoid aggregation phenomena, which is a major drawback.

Recent years have witnessed a very high level of research interest in well-defined biomimetic compounds. Because the micro/nanomaterials in nature usually possess intrinsic hydrophobicity and high adhesion, probe molecules can concentrate in a very small area after evaporation, resulting in relatively high SERS sensitivity [19]. Using the superhydrophobicity of the textured Taro leaf, Kumar and co-workers fabricated a novel SERS substrate and achieves a LOD value of 10^−11^ M for malachite green (MG) [20]. Likewise, Chamuah et al. employed cleaned diatom frustules as bio-templates for the self-assembly of Au nanoparticles that, when used as a SERS-active substrate, provided a LOD of 1 nM for MG with a relative standard deviation (RSD) value of 7.57% [21]. Pannico and co-workers proved that it was cost-effective and time-efficient to apply diatom frustules as a template for SERS-based investigations [22]. Meanwhile, natural wings of insects such as butterfly wing [23], cicada wing [24] and some special shells [25] are also known to comprise periodic and large-scale micro/nanostructures. Notably, a novel 3D biomimetic SERS substrate with a high density of “hot spots” was formed on a template of cicada wings via a simple one-step and reagent-free direct-current ion sputtering techniques by Prof. Han’s group [26]. These 3D plasmonic nanostructures are adopted because these 3D substrates offer a great deal of “hot spots” and binding sites for probe molecules within the laser illumination spot [27]. Cicada wings were used in their experiment because they comprise periodic and large-scale micro/nanostructures. They were applied in the label-free detection and sensing of animal viruses. Later, via a synchronous reduction process, Zhang et al. [28] successfully synthesized metallic replicas of a 3D butterfly wing array. They demonstrated that by further tuning the experimental parameters, a higher SERS performance could be achieved. With the significant progress in SERS, there emerges a pressing need for a facile and low-cost approach to fabricate hierarchical SERS systems with high sensitivity, stability and good reproducibility.

**Figure 1:**
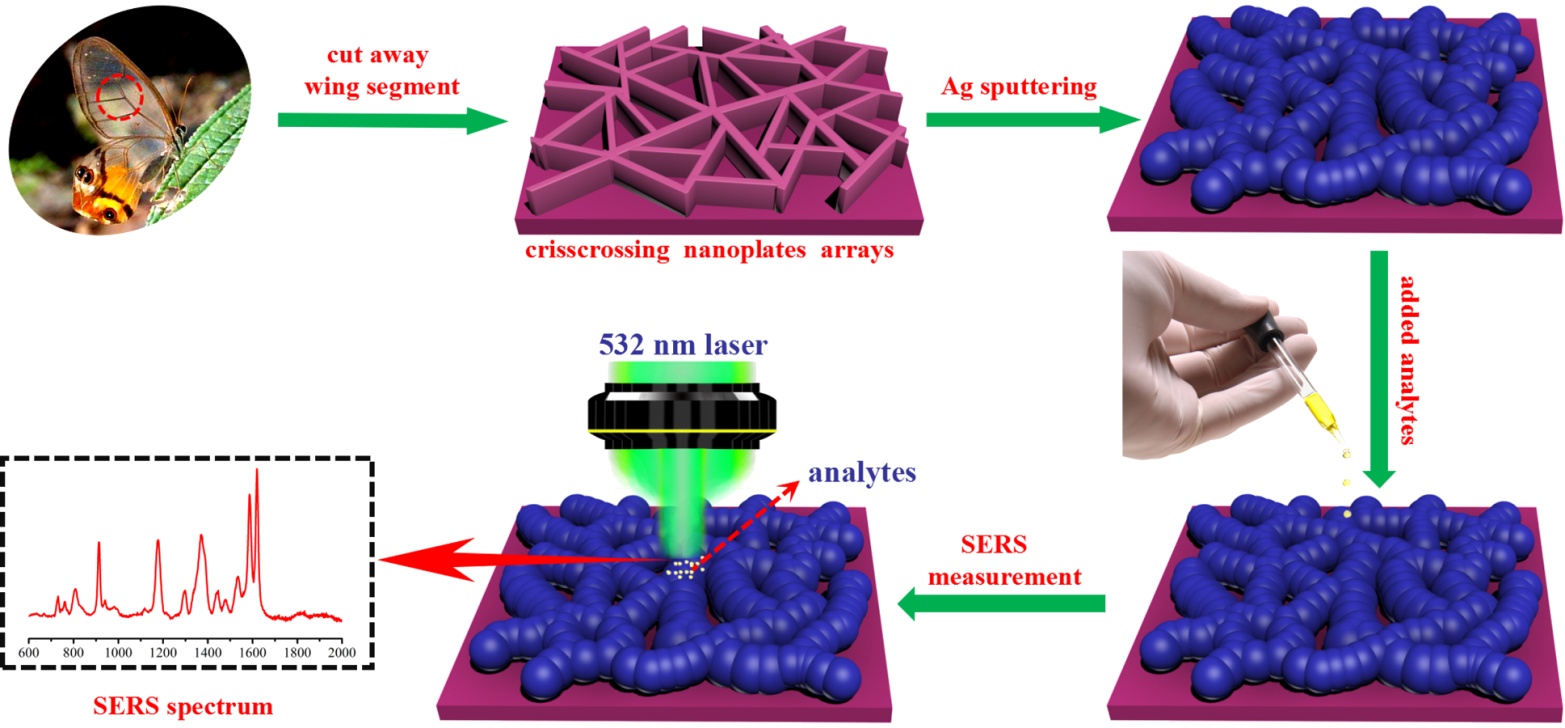
Schematic illustration of the fabrication process of the Ag-G.b. SERS substrate and SERS measurement by Raman system.

In this paper, with the aim to further improve the sensitivity and antioxidant properties as well as to achieve a high reproducibility of the substrates mentioned in our previous reports [29–30]. We adopt a highly efficient route (as shown in Figure 1) using biomimetic synthesis to fabricate 3D Ag nanofilm/glasswing butterfly wing (Ag-G.b.) hybrids as SERS substrates. The wings of the glasswing butterfly (*Haetera piera*) have an interesting nanostructure that can serve as a bio-scaffold for the coating with magnetron-sputtered Ag nanofilms. The thickness of Ag nanofilms and the size of the nanogaps deposited on the G.b. wing arrays can be easily controlled by tuning the sputtering time. In order to find the optimum SERS enhancement performance, the sputtering time is set to 5, 10, 15, 20 and 25 min, accordingly, and the SERS substrates are denominated Ag-G.b.-5, Ag-G.b.-10, Ag-G.b.-15, Ag-G.b.-20 and Ag-G.b.-25, respectively. G.b. wing arrays with 20 nm Ag nanofilms have the highest enhancement efficiency (EF = 2.96 × 10^7^) and higher SERS sensitivity to crystal violet (CV) with a limit of detection (LOD) of 10^−11^ M. The 3D finite-difference time-domain (3D-FDTD) simulation results suggest that the simulated electromagnetic field enhancement of Ag-G.b.-20 is close to the experimental value. Meanwhile, the Ag-G.b.-20 nanohybrids exhibited good stability and excellent reproducibility across the entire area with an average RSD value of less than 10.78%. Owing to the excellent sample collection efficiency, the proposed Ag-G.b.-20 substrate is employed to detect and quantify residues of the pesticide acephate, in rapid sampling and on-site chemical and environmental sensing.

## Results and Discussion

### Morphology characterization

**Figure 2:**
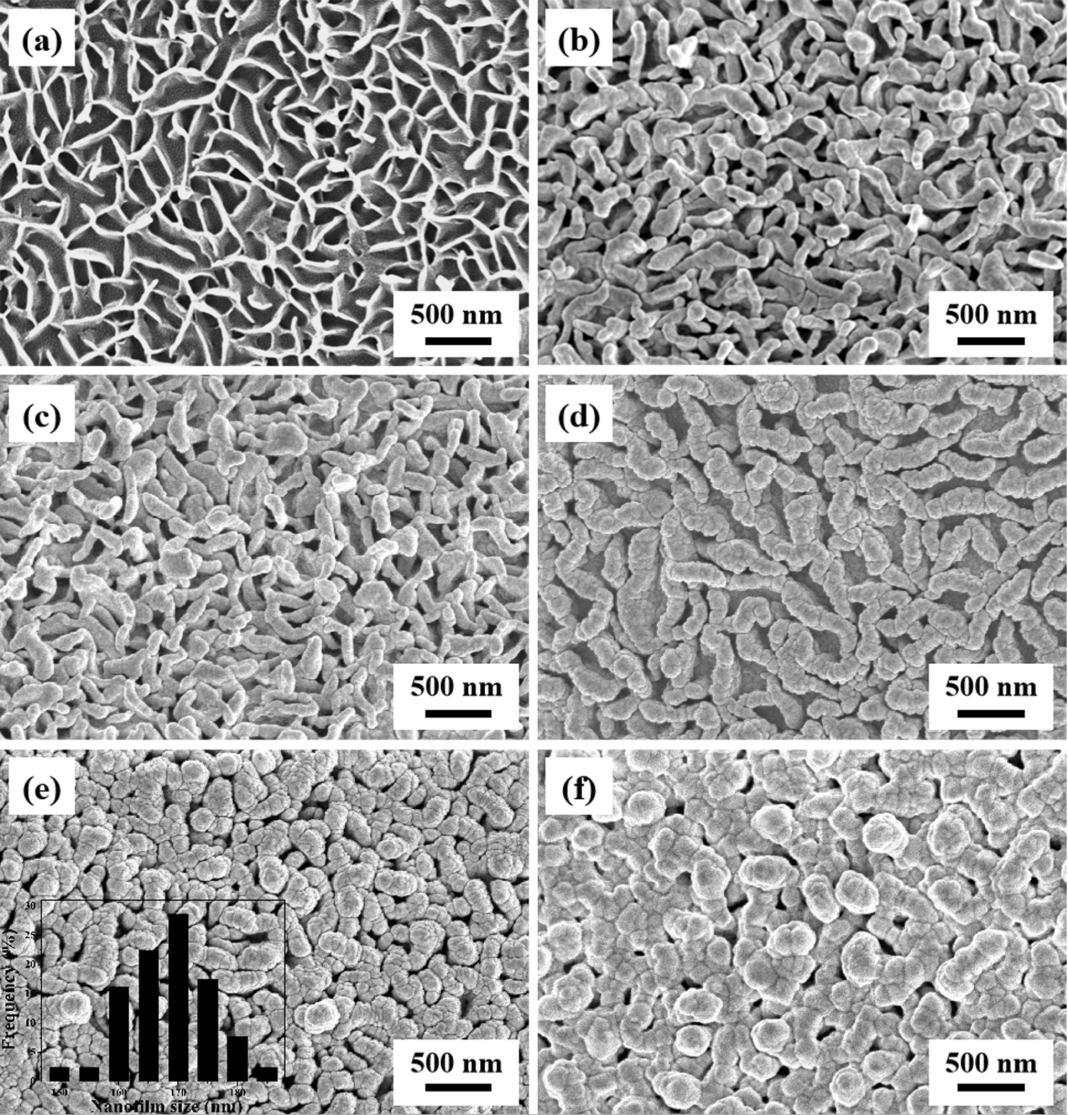
(a) Typical FE-SEM image of G.b.; FE-SEM images of Ag-G.b. substrates obtained with different sputtering time: (b) Ag-G.b.-5 substrate, (c) Ag-G.b.-10 substrate, (d) Ag-G.b.-15 substrate and (e) Ag-G.b.-20 substrate (the inset is the histograms of nanofilm size distribution of the Ag), (f) Ag-G.b.-25 substrate, respectively.

The morphology of neat G.b. wings was characterized by FE-SEM as shown in Figure 2a. We can observed that the surface of G.b. wings consists of interlaced vertical nanoplates with an average width of 30 ± 5 nm and an average length of 300 ± 20 nm. The rough nanostructures including the crisscrossing nanoplates are regarded as the origin of hydrophobicity. As shown in Figure 2b–f, Ag nanofilms were deposited on the surface of the G.b. wings and 3D plasmonic nanostructures with high EM were successfully fabricated. It can be clearly observed that Ag nanofilms successfully covered the crisscrossing nanoplates and extended along the vertical sides. At an initial sputtering time of 5 min, the average width of the Ag nanofilms was 50 ± 5 nm and the Ag nanofilms were grown uniformly on the nanoplates. In Figure 2c,d, the surface of G.b. wings is covered with thicker Ag nanofilms than the Ag-G.b.-5 substrate, with average widths of 70 ± 10 nm and 120 ± 10 nm, respectively. As shown in Figure 2e, after the sputtering time was increased to 20 min, the nanoplate-shaped nanostructures disappeared and nanorough Ag nanostructures were formed. Simultaneously, suitable nanogaps between the adjacent nanostructures were generated in which high near-field enhancement could be achieved. As shown in the inset of Figure 2e, where the Nano Measurer 1.2 software was used, the average width of the nanofilm was approximately 170 ± 10 nm (the average width was calculated based on 80 independent measurements on a Ag-G.b.-20 substrate). After further extension of the sputtering time to 25 min, larger and irregular Ag nanostructures were formed and the surface of G.b. became relatively smooth. These discoveries suggest that the width of Ag nanofilms and interfilm nanogaps can be easily controlled by adjusting the sputtering time. In addition, they reveal that our physical fabrication method could yield large-scale Ag nanofilms, unlike the synchronous reduction process reported in the previous report [28].

### SERS sensitivity of 3D Ag-G.b. hybrid composites

**Figure 3:**
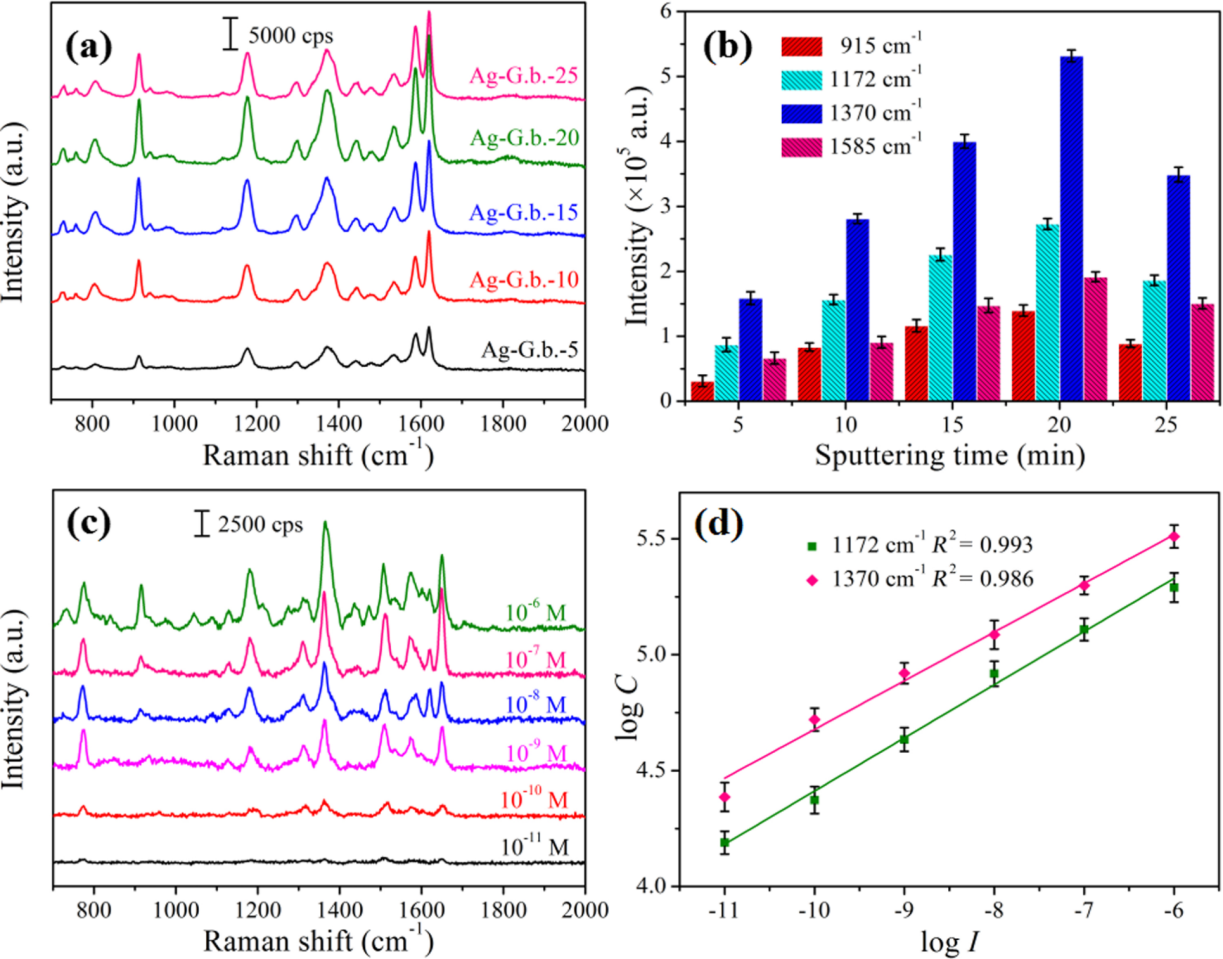
(a) Raman spectra of a 10^−4^ M CV solution absorbed on Ag-G.b.-5, Ag-G.b.-10, Ag-G.b.-15, Ag-G.b.-20 and Ag-G.b.-25 substrates. (b) Comparison of the CV Raman intensities centered at 915 cm^−1^, 1172 cm^−1^, 1370 cm^−1^ and 1585 cm^−1^ peaks with concentration of 10^−4^ M in different SERS-active substrates, respectively. (c) Raman spectra of CV at different concentrations from 10^−6^ M to 10^−11^ M on Ag-G.b.-20 substrate. (d) Linear calibration plot between the Raman intensities and CV concentrations in the log–log scale at the 1172 cm^−1^ and 1370 cm^−1^ peaks (the error bars were calculated based on five independent measurements).

The Ag nanofilm thickness and the hierarchical nanogaps of 3D Ag-G.b. are the key parameters that can influence the enhancement of SERS. Therefore, we used CV as probe molecule to evaluate the SERS performance of 3D Ag-G.b. with different Ag nanofilm thicknesses. Figure 3a shows the Raman spectra of 10^−4^ M CV adsorbed on 3D Ag-G.b. substrates with different Ag sputtering time. The characteristic peaks at 802, 915, 1172 and 1370 cm^−1^ correspond to C–H out-of-plane deformation, ring skeletal vibrations, C–H bending vibrations and C–C–C ring in-plane bending, respectively. The bands at 1532, 1585 and 1619 cm^−1^ were assigned to the ring C–C stretching [31–32]. Looking at the characteristic peak intensities shown in Figure 3b, we observed that within the first 20 min, the Raman intensities increased with the increase of sputtering time. This SERS performance improvement can be attributed to 3D plasmonic nanostructures formed by the Ag nanofilms sputtered onto the surface of G.b. wing arrays. When the sputtering time was more than 20 min, we observed an obvious decline of the Raman signal intensity. We chose the peak at 1370 cm^−1^ for illustration: The integrated Raman intensities of the Ag-G.b.-20 substrate at this peak were 3.35-times, 1.89-times, 1.33-times and 1.52-times higher than that of the Ag-G.b.-5, Ag-G.b.-10, Ag-G.b.-15 and Ag-G.b.-25 substrates, respectively. The Raman signal intensity of the Ag-G.b.-5 substrate was the lowest. This low intensity was due to the small quantity of Ag nanoparticles, which yielded a weak signal response compared to those of Ag-rich nanostructures. Due to the LSPR coupling effects, the Ag-G.b.-20 substrate with redundant Ag nanofilms showed the strongest Raman signal intensity.

Subsequently, a series of Raman spectra of CV with different concentrations (10^−11^ M to 10^−6^ M, aqueous solution) adsorbed on the optimal Ag-G.b.-20 SERS substrates were measured to demonstrate the sensitivity. From Figure 3c, the Raman spectral intensities decreased as the concentration of CV decreased. The characteristic peaks of CV could be evidently identified even at the ultra-low concentration of 10^−11^ M, which further revealed that the Ag-G.b.-20 substrate was highly sensitive with a LOD of 10^−11^ M for CV, which is promising for the application in chemical sensing and food security. Linear relationships between the integrated Raman intensities of two characteristic peaks (1172 cm^−1^ and 1370 cm^−1^) and the concentrations of CV were plotted in the log–log scale in Figure 3d. Satisfactory linear relationships (as shown in Table 1) could be found in the concentration range of 10^−11^ M to 10^−6^ M. As a good linear relationship is found in the low concentration range, we speculate that a thin layer of molecules (submonolayer to monolayer thickness) is adsorbed on the surface of the Ag-G.b.-20 substrate [33]. In the range of relatively low concentration, because all the probe molecules of CV are directly (or closely) adsorbed on the Ag-G.b.-20 substrate surface and SERS exhibits a proximity effect, the Raman signal intensities increase sharply as the probe molecule concentrations increase. In additon, Qin et al. [34] showed that in the high-concentration region (10^−5^ M to 10^−3^ M), the linear relationships were piecewise due to multilayer adsorption.

**Table 1:** Linear relationships between CV concentrations (10^−6^ to 10^−11^ M) and Raman intensities at characteristic peaks of CV.

wave number/cm^−1^	linear function	correlation coefficient (*R*^2^)	*n*

1172	y = 0.229x + 6.706	0.993	6
1370	y = 0.210x + 6.779	0.986	6

### EF calculation and 3D finite-difference time-domain simulation

To better evaluate the SERS enhancement performance of the Ag-G.b.-20 substrates, an enhancement factor (EF) has been estimated based on Equation 1 [1,20]:

[1] $$\text{EF}=\frac{I_{\text{SERS}}}{I_{\text{bulk}}}\times \frac{N_{\text{bulk}}}{N_{\text{SERS}}}$$

where the *I*_SERS_ and *I*_bulk_ are the integrated intensities of a same Raman peak in the SERS spectrum and bulk Raman spectrum, respectively. The *N*_SERS_ and *N*_bulk_ denote the numbers of the CV molecules effectively excited by a laser beam on the corresponding substrates. In this calculation, 10^−2^ M of CV solution was used for the conventional Raman measurement, and 10 μL of 10^−6^ M CV solution was dripped onto the Ag-G.b.-20 substrate for SERS detection. Figure S1a,b in Supporting Information File 1 shows the SERS spectrum of 10^−6^ M of CV solution obtained from the Ag-G.b.-20 substrate and the conventional Raman spectrum of 10^−2^ M CV solution. The integrated Raman intensities *I*_SERS_ and *I*_bulk_ for 1370 cm^−1^ peak were 3.25 × 10^5^ and 396.32, respectively. Therefore, the ratio of *I*_SERS_*/I*_bulk_ was calculated to be 2052.80. The *N*_bulk_ was calculated according to Equation 2:

[2] $$N_{\text{bulk}}=A\times h\times c_{\text{bulk}}\times N_{\text{A}}$$

where *A* is the laser spot area, *h* is the laser penetration depth, *c*_bulk_ is the concentration of the CV solution and *N*_A_ is the Avogadro constant. In the Raman measurement, the diameter of the illumination spot was ca. 1 µm and the penetration depth was ca. 3 mm. Hence, the value of *N*_bulk_ was ca. 1.42 × 10^10^. According to the above analysis, when the concentration of the CV was 10^−6^ M, the CV molecules were absorbed as a monolayer on the surface of the Ag-G.b.-20 substrate. The surface area of a single CV molecule is ca. 2.0 nm^2^ [35]. Afterwards, dividing the area of the laser spot (0.785 µm^2^) by 2.0 nm^2^, the calculated *N*_SERS_ equals 3.93 × 10^5^. According to the above calculation, the EF for the Ag-G.b.-20 substrate was calculated to be 2.96 × 10^7^.

**Figure 4:**
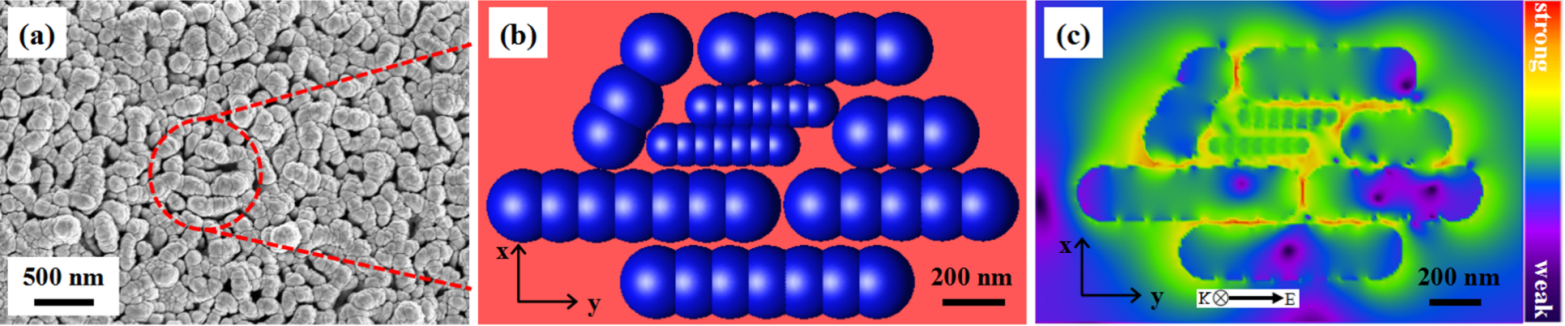
(a) FE-SEM images of Ag-G.b.-20 SERS substrate (the region in the red circle is the selected area used in the 3D-FDTD simulation). (b) Simulation model Ag-G.b.-20 SERS substrate. (c) The spatial distribution of the electromagnetic field intensity simulation results.

Based on the arrangement of Ag nanofilms on the G.b. template, we also studied the electromagnetic field distribution in the vicinity of the Ag-G.b.-20 plasmonic nanostructures using the 3D finite-difference time-domain (3D-FDTD) method. Considering that Ag can be described by the Debye–Drude model, the Yee cell in our calculation was set to 1 nm × 1 nm × 1 nm. The dielectric function of a metal is a complex entity as a function of the wavelength. Therefore, the infinite-frequency relative dielectric constant and the zero-frequency relative dielectric constant of Ag were set according to the Gai’s work [36]. In order to simulate as close as possible to the experimental results, the simulation model of the nanostructure was extracted from FE-SEM images (Figure 4a). Figure 4b is the corresponding simulation model and a square wave with a wavelength of 532 nm was simulated to illuminate the nanostructure along the *z*-direction. The width of the Ag nanofilms coated on the substrate was considered to be 200 nm and the length of the nanostructures was assumed to vary between 400 to 700 nm. Figure 4c shows the simulated electromagnetic field distribution of the Ag-G.b.-20 plasmonic nanostructures. We observed a maximum electromagnetic field magnitude of 57.30 V/m in the nanostructure, the calculated EF of which equals 1.07 × 10^7^ (Supporting Information File 1 for details). From the electromagnetic field distribution in the Figure 4c, we can conclude that the Ag-G.b.-20 SERS substrate generates LSPR and the large-scale “hot spots” are localized in the narrow nanogaps where the electromagnetic field enhancement occurs.

### Long-term stability and SERS reproducibility of the Ag-G.b.-20 SERS substrates

**Figure 5:**
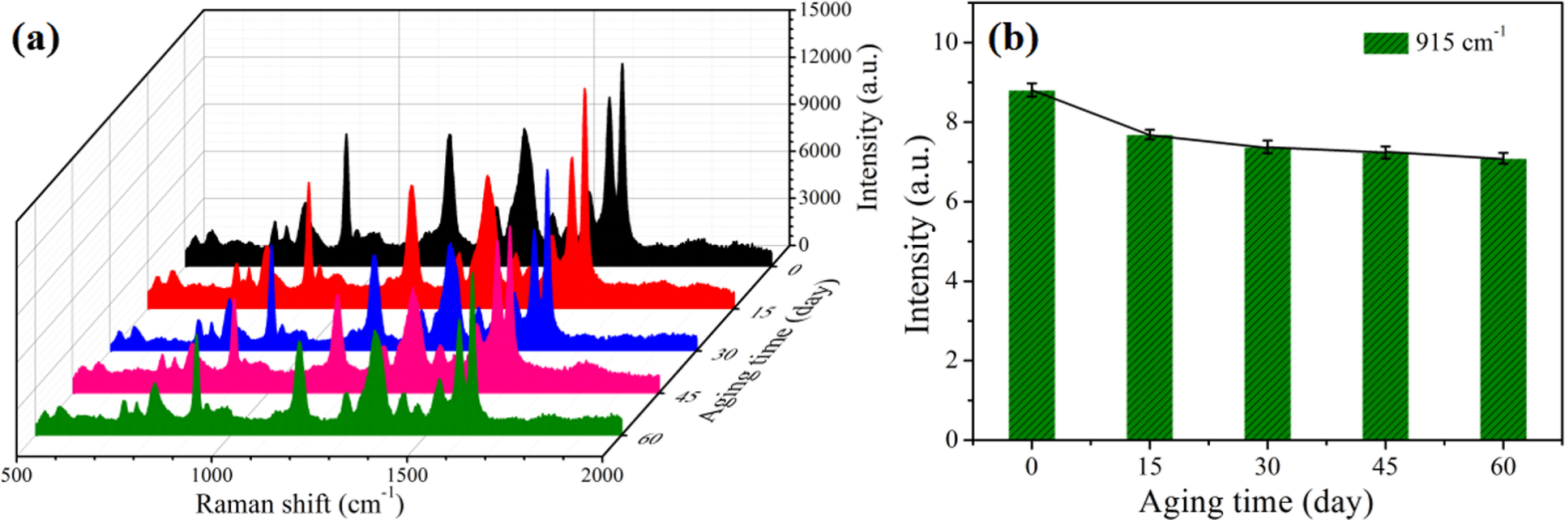
(a) Raman spectra of 10^−4^ M CV adsorbed on Ag-G.b.-20 substrates after different aging times. (b) Plot of Raman intensities of CV at 915 cm^−1^ as a fucntion of the aging time (the error bars were calculated based on five independent measurements).

In addition to high SERS enhancement and sensitivity, long-term stability is another important parameter for an ideal SERS substrate. Therefore, the long-term stability of Ag-G.b.-20 substrates was examined under ambient conditions. Figure 5a shows the Raman spectra of 10^−4^ M CV recorded on the Ag-G.b.-20 substrates and the corresponding substrates stored for 15, 30, 45 and 60 days. Figure 5b clearly reveals that the integrated Raman intensity at 915 cm^−1^ decreased after the initial 15 days (about 12.70%) and then the signal intensities tended to level off. After aging for 60 days, the Raman signal intensity decreased by 19.47% (70921 to 88064). These results indicate that the Ag-G.b.-20 substrates can be used in practical application and further demonstrate that the Ag nanofilms are firmly attached to the G.b. wing substrates.

**Figure 6:**
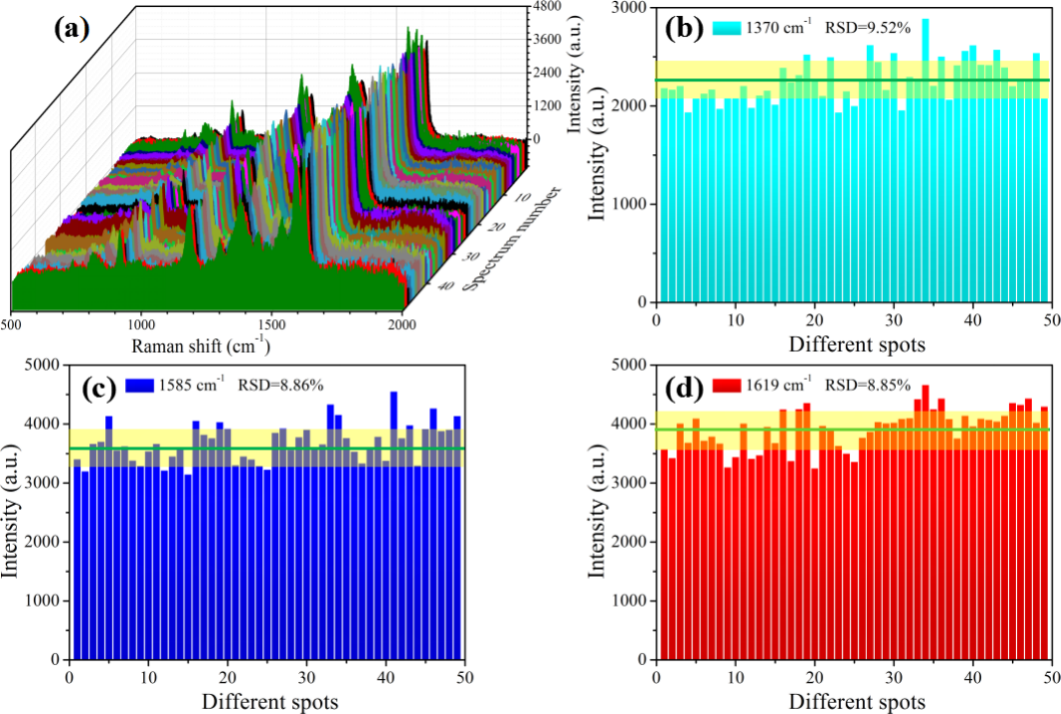
(a) A series of Raman spectra of 10^−7^ M CV solution collected from 49 randomly selected spots of the optimal Ag-G.b.-20 substrates. (b–d) The main Raman intensities of 10^−7^ M CV at characteristic Raman peaks (b: 1370 cm^−1^; c: 1585 cm^−1^; d: 1619 cm^−1^) and the corresponding RSD values.

The Raman signal reproducibility is of great significant for a SERS substrate. Previous studies have well established that the “hot spots” of periodic distribution in the SERS substrates determine the reproducibility of Raman signals. The FE-SEM image (Figure 2e) shows that the Ag nanostructures do not uniformly arrange on the G.b. wing surface, and the diameter of the laser spot was 1 μm in the Raman measurement. Therefore, the relative standard deviation (RSD) was introduced to evaluate the signal reproducibility of the as-prepared Ag-G.b.-20 substrates [37]. Here, 10 μL of 10^−7^ M CV solution was dropped on seven Ag-G.b.-20 substrates, and 49 Raman spectra, which were randomly selected from the pre-treated seven samples, were recorded (Figure 6a). The main Raman bands of CV showed no significant shift or change in Raman intensity and the RSD values for characteristic the peaks at 1370, 1585 and 1619 cm^−1^ were calculated to be 9.52%, 8.86% and 8.85% respectively as shown in Figure 6b–d (details can be found in Supporting Information File 1), which further demonstrated the reproducibility of the measurements. To further demonstrate the point-by-point reproducibility, a Raman mapping with an area of 7 μm × 7 μm was conducted with 10^−7^ M CV solution on a Ag-G.b.-20 substrate. The Raman signal distribution at 1532 cm^−1^ is shown in Figure S2a, where the signal intensities are very uniform except for a few points. As plotted in Figure S2b, the RSD values for 915, 1172 and 1532 cm^−1^ were estimated to be 10.78%, 8.91% and 9.25%. Based on above results, both substrate-by-substrate and point-by-point reproducibility can be achieved with Ag-G.b.-20 substrates, which is likely connected to the monolayer adsorption of CV molecules and the large-scale distribution of “hot-spots” across the entire surface of the Ag-G.b.-20 substrates. In all, the essential performances of Ag-G.b.-20 substrate, such as EF, sensitivity, reproducibility and stability, are superior to those of substrates in the previous studies [29–30].

### Application of the Ag-G.b.-20 SERS substrates for acephate detection

**Figure 7:**
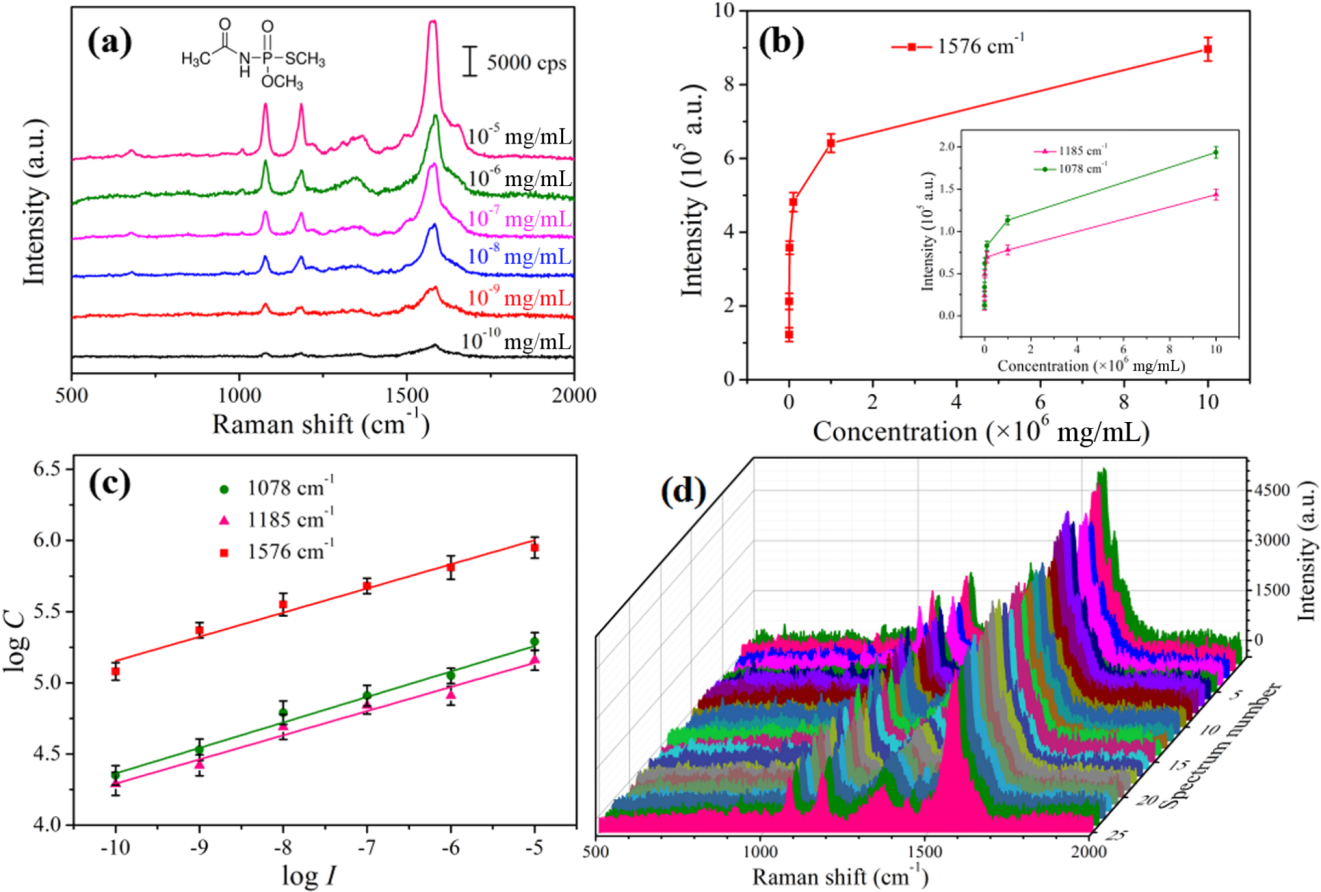
(a) Raman spectra of acephate with the concentrations of 10^−5^ to 10^−10^ mg/mL. (b) Corresponding Raman intensities of the main peaks at 1078, 1185 and 1576 cm^−1^ in different concentrations. (c) Linear calibration plot between the Raman intensities and acephate concentrations. (d) Raman spectra of 10^−8^ mg/mL acephate collected by using the Ag-G.b.-20 substrate.

The residues of organophosphorus pesticides pose a serious threat to public health and food safety. Hence, the sensitive detection of organophosphorus pesticides in vegetables and fruits has attracted wide attention. Among the reported methods of pesticide residue detection [38–40], SERS is an accurate, rapid and non-destructive spectral analysis technique that can take advantage of “fingerprint” information to characterize the target analytes. Here, we evaluated the practicability of the Ag-G.b.-20 SERS substrate as a high-throughput and efficient SERS substrate in the detection of acephate. After pre-treatment, 10 μL acephate ethanol solutions with different concentrations were adsorbed on the Ag-G.b.-20 SERS substrates, followed by SERS detection after drying. The Raman spectra of acephate obtained by Ag-G.b.-20 substrates were shown in Figure 7a. A sensitivity to acephate with a LOD as low as 10^−10^ mg/mL was achieved. Comparing with the limit of quantification (LOQ) on apples according to the National food safety standard (GB 2763-2014) in China (Table 2), the LOD of Ag-G.b.-20 SERS substrate is far less than the LOQ. Thus, the substrate is a perfect candidate for the application in pesticide detection. Meanwhile, as shown in Figure 7b, as the concentrations of acephate decreased, the characteristic peak intensities gradually decreased. Figure 7c shows the linear correlations of the Raman peaks at 1078, 1185 and 1576 cm^−1^ as functions of the concentration in the log–log scale. The linear equation and *R*^2^ values for three peaks are given in Supporting Information File 1, Table S1. The high-efficiency detection of acephate molecule in ultra-low concentrations proved the applicability of the Ag-G.b.-20 SERS substrate for quantitative analysis. The reproducibility detection of pesticide molecules by Ag-G.b.-20 substrate was also conducted by employing 10^−8^ mg/mL acephate solution, and the Raman spectra recorded from 25 randomly selected points are shown in Figure 7d. The RSD values of 1185 and 1576 cm^−1^ were calculated to be 11.51% and 8.54%, respectively, as shown in Supporting Information File 1, Figure S3a and Figure S3b, revealing the high reproducibility of Ag-G.b.-20 substrate measurements.

**Table 2:** Analytical figures of merit for the quantitative SERS detection of acephate (The concentration converted from mass-to-area ratio to mg per kilogram was roughly calculated according to the reported work [41] and the detailed explanation in Supporting Information File 1).

limit of detection (LOD)	limit of quantification (LOQ)

1 × 10^−10^ mg/mL ≈ 2 × 10^−6^ ng/cm^2^	0.625 ng/cm^2^

## Conclusion

In summary, the 3D Ag nanofilms/glasswing butterfly wing biomimetic arrays have been successfully fabricated by magnetron sputtering. The thickness of the Ag nanofilms can be well controlled by adjusting the sputtering time. The Ag-G.b.-20 substrate with large-scale “hot spots” and an increased roughness shows the highest enhancement efficiency for CV molecules. The EF for Ag-G.b.-20 arrays is up to 2.96 × 10^7^. Also, the Ag-G.b.-20 substrate showed a superior SERS detection sensitivity with a LOD as low as 10^−11^ M. In addition, the RSD values for CV are less than 10.78%, demonstrating the outstanding reproducibility of the substrate. The Ag-G.b.-20 substrates still show a good stability of the Raman signal after aging for 60 days. Owing to the high performance of the novel SERS substrate, the trace detection of acephate is successfully achieved by using Ag-G.b.-20 arrays with a LOD of 10^−10^ mg/mL. We envision that the designed 3D biomimetic SERS substrate can potentially emerge as a candidate for rapid and reliable trace detection of other chemical, biological and hazardous samples.

## Experimental

### Materials

The glasswing butterflies (*Haetera piera diaphana*) were purchased from Beijing Jiaying Grand Life Sciences Co., Ltd. (Beijing, China). Experiments with glasswing butterflies complied with the accepted ethical standards and were approved by the Ethical Review Board of Yanshan University on 15 January 2018. Crystal violet was supplied by Key Laboratory for Microstructural Material Physics of Hebei Province. Acephate and ethanol were of analytical grade and obtained from J&K Scientific Ltd. (Beijing, China). The silver target (diameter: 50.8 mm, thickness: 3.175 mm, purity: 99.99%) was obtained from China Material Technology Co., Ltd. (Jiangxi, China). All the chemicals were used without further purification. Deionized water (18.25 MΩ·cm) was used throughout all the experiments.

### Sample preparation

Before preparing the samples, the glasswing butterfly wings were first cleaned in deionized water for 10 min to remove the adsorbed contaminants. Afterwards, the transparent regions were cut out as templates for SERS-active substrates. Ag nanofilms were sputtered on the G.b. wings by radio-frequency magnetron sputtering system (DHRM-3). The pressure of the sputtering chamber was set to be 3.5 × 10^−3^ Pa, the sputtering voltage was 90 V and the current was 170 mA.

### Characterization and SERS measurements

The morphologies of the G.b. wings before and after coating were characterized by field-emission scanning electron microscopy (FE-SEM, JEOL JSM-2100). For SERS measurements a Renishaw inVia Raman system was employed. This system was combined with a microscope objective (50× objective, numerical aperture (NA) = 0.75) to focus the laser on the substrates. Under excitation wavelength of 532 nm, the diameter of the laser spot was about 1 μm, the spectral resolution was 1 cm^−1^, the power was kept at 0.5 mW, the exposure time was 10 s and the number of accumulations for each measurement was two. For each substrate, 10 μL CV (10^−4^ to 10^−11^ M) was added directly on the different prefabricated SERS substrates and let to dry at room temperature to achieve the adsorption of as many CV molecules as possible on the surface of the Ag nanofilms. The detection procedure of different concentrations of acephate (10^−5^ to 10^−10^ mg/mL, ethanol solution) was consistent with that of the CV solutions. In order to obtain the statistical results, the Raman spectra (except given otherwise) were taken from five different area of each sample.

## Supporting Information

1. Enhancement factor (EF) calculation; 2. The equation which was used to calculate the EF; 3. The equation which was used to calculate the RSD; 4. Point-by-point reproducibility of Ag-G.b.-20 substrate; 5. The concentration converted from mg-to-mL to mass-to-area ratio; 6. RSD values of 1185 and 1576 cm^−1^ of acephate.

File 1 Additional theoretical and experimental information.

## References

[1] Stiles P L, Dieringer J A, Shah N C, Van Duyne R P (2008). Annu Rev Anal Chem.

[2] Le Ru E C, Etchegoin P G (2012). Annu Rev Phys Chem.

[3] Willets K A, Van Duyne R P (2007). Annu Rev Phys Chem.

[4] Li X, Chen G, Yang L, Jin Z, Liu J (2010). Adv Funct Mater.

[5] Chamuah N, Bhuyan N, Das P P, Ojah N, Choudhary A J, Medhi T, Nath P (2018). Sens Actuators, B.

[6] Campion A, Kambhampati P (1998). Chem Soc Rev.

[7] Sivashanmugan K, Liao J-D, Yao C-K (2014). Appl Phys Express.

[8] Zheng X-S, Jahn I J, Weber K, Cialla-May D, Popp J (2018). Spectrochim Acta, Part A.

[9] Vianna P G, Grasseschi D, Costa G K B, Carvalho I C S, Domingues S H, Fontana J, de Matos C J S (2016). ACS Photonics.

[10] Zhu J, Liu M-J, Li J-J, Li X, Zhao J-W (2018). Spectrochim Acta, Part A.

[11] Banchelli M, Tiribilli B, de Angelis M, Pini R, Caminati G, Matteini P (2016). ACS Appl Mater Interfaces.

[12] Zhang Q, Large N, Nordlander P, Wang H (2014). J Phys Chem Lett.

[13] Chen S Y, Liu B, Zhang X Y, Mo Y L, Chen F, Shi H, Zhang W H, Hu C L, Chen J (2018). Electrochim Acta.

[14] Brolo A G, Arctander E, Gordon R, Leathem B, Kavanagh K L (2004). Nano Lett.

[15] Yue W S, Yang Y, Wang Z H, Han J G, Syed A, Chen L Q, Wong K C, Wang X B (2012). J Phys D: Appl Phys.

[16] Nien L-W, Chao B-K, Li J-H, Hsueh C-H (2015). Plasmonics.

[17] Zhang J, Irannejad M, Cui B (2015). Plasmonics.

[18] Sivashanmugan K, Liao J-D, Liu B H, Yao C-K, Luo S-C (2015). Sens Actuators, B.

[19] Wang J, Zheng Y, Nie F-Q, Zhai J, Jiang L (2009). Langmuir.

[20] Kumar P, Khosla R, Soni M, Deva D, Sharma S K (2017). Sens Actuators, B.

[21] Chamuah N, Chetia L, Zahan N, Dutta S, Ahmed G A, Nath P (2017). J Phys D: Appl Phys.

[22] Pannico M, Rea I, Chandrasekaran S, Musto P, Voelcker N H, De Stefano L (2016). Nanoscale Res Lett.

[23] Nishimoto S, Bhushan B (2013). RSC Adv.

[24] Mo X, Wu Y, Zhang J, Hang T, Li M (2015). Langmuir.

[25] Lu Z Y, Liu Y J, Wang M H, Zhang C, Li Z, Huo Y Y, Li Z, Xu S C, Man B Y, Jiang S Z (2018). Sens Actuators, B.

[26] Shao F, Lu Z C, Liu C, Han H Y, Chen K, Li W T, He Q G, Peng H, Chen J N (2014). ACS Appl Mater Interfaces.

[27] Kodiyath R, Papadopoulos T A, Wang J, Combs Z A, Li H, Brown R J C, Brédas J-L, Tsukruk V V (2012). J Phys Chem C.

[28] Zhang M F, Meng J T, Wang D P, Tang Q, Chen T, Rong S Z, Liu J Q, Wu Y C (2018). J Mater Chem C.

[29] Wang Y H, Wang M L, Shen L, Sun X, Shi G C, Ma W L, Yan X Y (2018). Appl Surf Sci.

[30] Wang Y H, Wang M L, Sun X, Shi G C, Zhang J Z, Ma W L, Ren L J (2018). Opt Express.

[31] Cañamares M V, Chenal C, Birke R L, Lombardi J R (2008). J Phys Chem C.

[32] Sancı R, Volkan M (2009). Sens Actuators, B.

[33] Cheng H-W, Huan S-Y, Wu H-L, Shen G-L, Yu R-Q (2009). Anal Chem (Washington, DC, U S).

[34] Chen J, Qin G W, Shen W, Lic Y Y, Das B (2015). J Mater Chem C.

[35] Kudelski A (2005). Chem Phys Lett.

[36] Gai H F, Wang J, Tian Q (2007). Appl Opt.

[37] Parsons H M, Ekman D R, Collette T W, Viant M R (2009). Analyst.

[38] Omar N, Bakar J, Muhammad K (2013). Food Control.

[39] Hou J Y, Dong J, Zhu H S, Teng X, Ai S Y, Mang M L (2015). Biosens Bioelectron.

[40] Schieberle P, Molyneux R J (2012). J Agric Food Chem.

[41] Liu B H, Han G M, Zhang Z P, Liu R Y, Jiang C L, Wang S H, Han M-Y (2012). Anal Chem (Washington, DC, U S).

