# Supplementary material for: Biomimetic synthesis of Ag-coated glasswing butterfly arrays as ultra-sensitive SERS substrates for efficient trace detection of pesticides
Source: Beilstein J Nanotechnol. 2019 Feb 28;10:578–88. doi: 10.3762/bjnano.10.59 (PMC6404425; doi:10.3762/bjnano.10.59)
Supplement: File 1 — Additional theoretical and experimental information. [file Beilstein_J_Nanotechnol-10-578-s001.pdf]

BEILSTEIN JOURNAL OF NANOTECHNOLOGY

Supporting Information

for

Biomimetic synthesis of Ag-coated glasswing butterfly arrays as ultra-sensitive SERS substrates for efficient trace detection of pesticides

Guochao Shi, Mingli Wang, Yanying Zhu, Yuhong Wang, Xiaoya Yan, Xin Sun, Haijun Xu and Wanli Ma

Beilstein J. Nanotechnol. **2019,** *10,* 578–588. doi:10.3762/bjnano.10.59

Additional theoretical and experimental information

License and Terms: This is a supporting information file under the terms of the Creative Commons Attribution License (http://creativecommons.org/licenses/by/4.0). Please note that the reuse, redistribution and reproduction in particular requires that the authors and source are credited.
The license is subject to the *Beilstein Journal of Nanotechnology* terms and conditions: (https://www.beilstein-journals.org/bjnano)

1 Enhancement factor (EF) calculation.

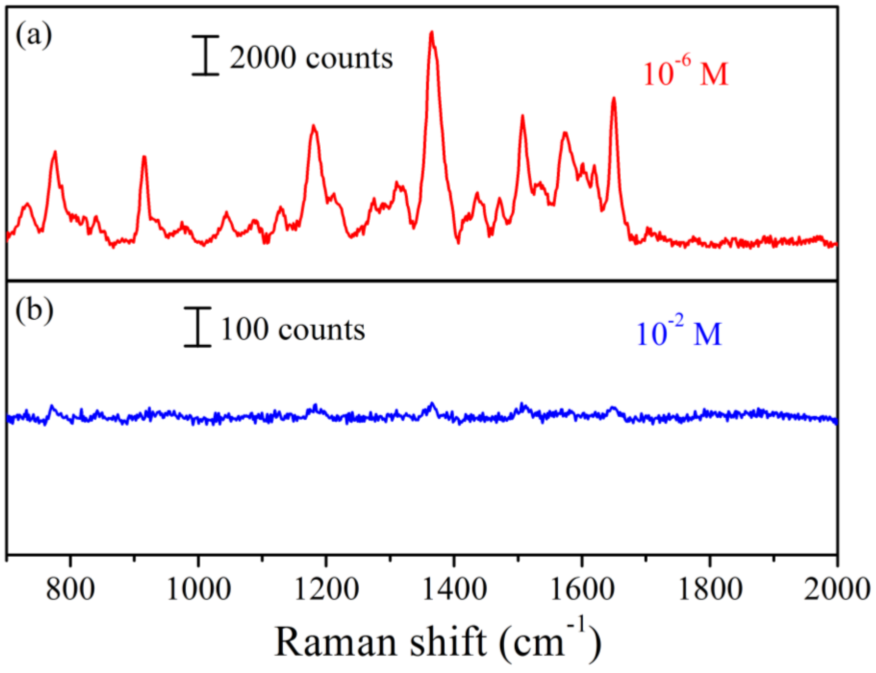

Figure S1: (a) SERS spectrum of 10^{-6} M CV solution acquired from Ag-G.b.-20 substrate. (b) Normal Raman spectrum of 10^{-2} M CV solution.

2 The calculated EF was by the following equation(1) [1]:

$$G_{SERS} = \left| \frac{E_{loc}(\omega)}{E_{inc}(\omega)} \right|^4 \quad (1)$$

where the $E_{loc}(\omega)$ and $E_{inc}(\omega)$ represent to the E and E_0 in the 3D-FDTD simulation, respectively.

3 The RSD value was calculated according to the following equation (2) [2]:

$$RSD = \frac{\sqrt{\frac{\sum_{i=1}^{n}(I_i - \bar{I})^2}{n-1}}}{\bar{I}} \quad (2)$$

where the $\bar{I}$ represents the average intensity of the Raman signal, n is the number of the measured spectrum, I_i is the Raman intensity of each spectrum at a characteristic peak.

4 Point-by-point reproducibility of Ag-G.b.-20 substrate.

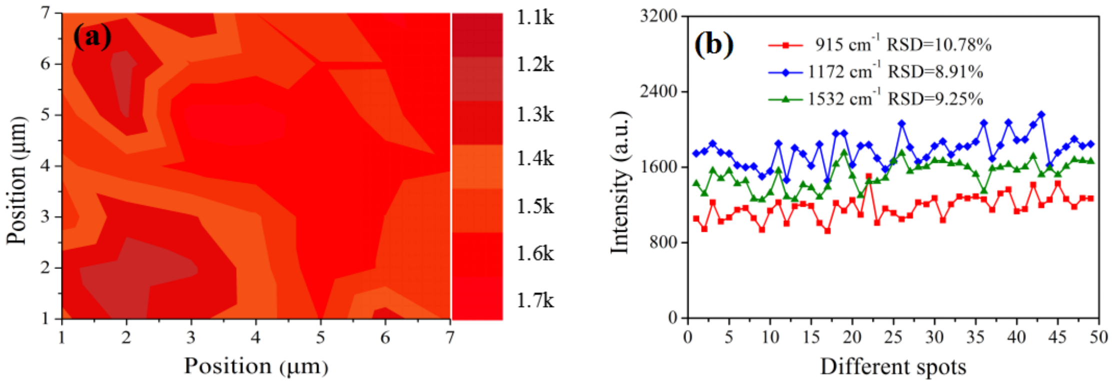

Figure S2: (a) Raman mapping of the 1532 cm^{-1} peak. (b) The RSD of Raman intensities of 915, 1172 and 1532 cm^{-1} (C_{CV}=10^{-7} M) for an area of 7 μm ×7 μm on a Ag-G.b.-20 substrate.

5 The concentration converted from mg-to-ml to mass-to-area ratio:

In our experiment, the detection of 10^{-10} mg/ml acephate was based on 10 μL acephate ethanol solution adsorbed on the Ag-G.b.-20 substrate with a cover area of approximately 0.5 cm^2. By unit conversion, 10^{-10} mg/ml was approximately equaled to 2×10^{-6} ng cm^{-2}. Meanwhile, the concentration converted from mass-to-area ratio to mg per kilogram was roughly calculated according to the reported work [3].

Table S1: Linear relationships between acephate concentrations (10^{-5} mg mL^{-1}-10^{-10} mg mL^{-1}) and Raman intensities at characteristic peaks of acephate.

Peak/cm^{-1}	Linear function	R^2
1078	y=0.179x+6.155	0.986
1185	y=0.170x+5.990	0.966
1576	y=0.170x+6.851	0.961

6 RSD values of 1185 and 1576 cm^{-1} of acephate.

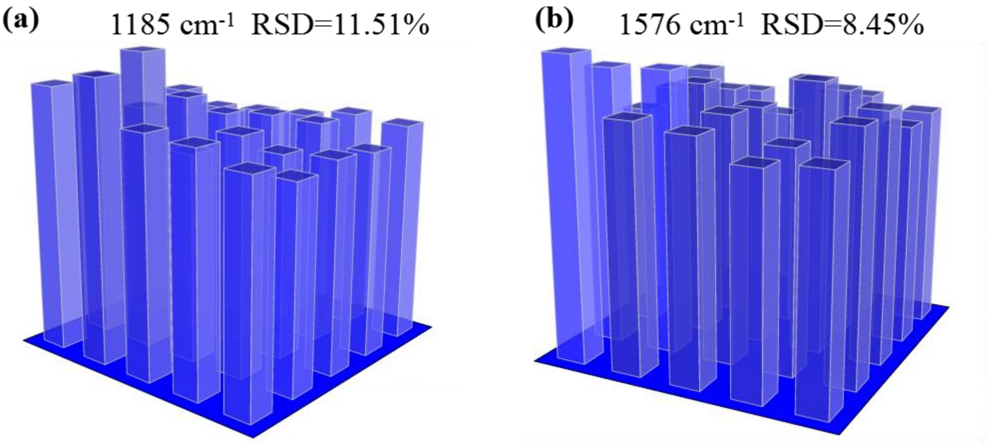

Figure S3: The corresponding intensity distributions at 1185 and 1576 cm^{-1} of 10^{-8} mg/ml acephate molecules from 25 Raman spectra.

References

1. García-Vidal, F. J.; Pendry, J. B. *Phys. Rev. Lett.* **1996**, 77, 1163-1166.
2. Parsons, H. M.; Ekman, D. R.; Collette, T. W.; Viant, M. R. *Analyst* **2009**, 134, 478-485.
3. Liu, B. H.; Han, G. M.; Zhang, Z. P.; Liu, R. Y.; Jiang, C. L.; Wang, S. H.; Han, M. Y. *Anal. Chem.* **2012**, 84, 255-261.
